# Clinical and socioeconomic burden of rhinoviruses/enteroviruses in the community

**DOI:** 10.1111/irv.12989

**Published:** 2022-04-29

**Authors:** Katia Camille Halabi, Melissa S. Stockwell, Luis Alba, Celibell Vargas, Carrie Reed, Lisa Saiman, The Mobile Surveillance for Acute Respiratory Infection/Influenza‐like Illness in the Community (MoSAIC) Study Team

**Affiliations:** ^1^ Columbia University Irving Medical Center New York New York USA; ^2^ Mailman School of Public Health Columbia University New York New York USA; ^3^ New York‐Presbyterian Hospital New York New York USA; ^4^ Centers for Disease Control and Prevention Atlanta Georgia USA; ^5^ Present address: Nationwide Children's Hospital The Ohio State University College of Medicine Columbus Ohio USA

**Keywords:** antibiotic stewardship, clinical burden, community surveillance, enterovirus, rhinovirus, socioeconomic burden

## Abstract

**Background:**

The epidemiology, clinical features, and socioeconomic burden associated with detection of rhinoviruses (RV)/enteroviruses (EV) from individuals in the community with acute respiratory infections (ARIs) are not fully understood.

**Methods:**

To assess the clinical and socioeconomic burden associated with RV/EV, a secondary analysis of data collected during a prospective, community‐based ARI surveillance study was performed. From December 2012 to September 2017, adult and pediatric participants with ARIs had nasopharyngeal specimens obtained and tested by multiplex polymerase chain reaction assay. Characteristics and socioeconomic burden including missed school or work and/or antibiotic use among participants who did and did not seek medical care and among participants with and without co‐detection of another respiratory pathogen with RV/EV were compared.

**Results:**

Throughout the study period, RV/EV was detected in 54.7% (885/1617) of ARIs with a respiratory pathogen detected. Most ARI episodes associated with RV/EV occurred in females (59.1%) and children ≤17 years old (64.2%). Those ≤17 years were more likely to seek medical care. Compared to those not seeking medical care (*n* = 686), those seeking medical care (*n* = 199) had a longer duration of illness (5 vs. 7 days) and were more likely to miss work/school (16.4% vs. 47.7%) and/or use antibiotics (3.6% vs. 34.2%). Co‐detection occurred in 8% of ARIs of which 81% occurred in children. Co‐detection was not associated with longer illness, more missed work/or school, or antibiotic use.

**Conclusion:**

Non‐medically attended and medically attended ARIs associated with RV/EV resulted in clinical and socioeconomic burden, regardless of co‐detection of other respiratory pathogens.

## 1 INTRODUCTION

Rhinoviruses (RV) and respiratory enteroviruses (EV) are leading causes of acute respiratory infections (ARIs).^1^, ^2^ Current diagnostic molecular techniques do not differentiate between RV and EV; however, recent epidemiological studies have demonstrated that RV is frequently detected in adults and pediatric patients with upper or lower respiratory tract infections.^2^ In adults, RV have been associated with exacerbation of chronic lung disease including asthma, chronic obstructive pulmonary disease, and cystic fibrosis.^2^ In children, RV/EV can cause severe illness^3^; rates of intensive care unit admission, mechanical ventilation, and supplemental oxygen are similar to these rates associated with influenza, respiratory syncytial virus (RSV), parainfluenza, and human metapneumovirus.^4^ In addition, RV/EV are associated with a socioeconomic burden resulting from both direct healthcare costs and indirect costs resulting from missed work or school.^1^ However, most studies have described the burden of RV/EV in hospitalized patients and in those who sought care.^5^, ^6^, ^7^ Community‐based studies, which include individuals who do and do not seek medical care, would further expand our understanding of the clinical and socioeconomic burden associated with RV/EV.

We assessed the burden of RV/EV using data from a prospective, community‐based, surveillance study of ARIs that aimed to study the epidemiology and impact of respiratory viruses.^8^ In this secondary analysis, we assessed the characteristics of participants who had ARIs with RV/EV detected and RV/EV seasonality during a 5‐year study period. We compared the demographic and clinical characteristics, symptoms, and the socioeconomic burden (defined as missed work or school or antibiotic use) among those who did and did not seek medical care. Due to the potential for RV/EV to be associated with prolonged shedding,^9^, ^10^ we also explored the impact of RV/EV alone versus RV/EV co‐detected with other respiratory viruses.

## 2 METHODS

### 2.1 Study design and study participants

This retrospective cohort study was a secondary analysis of participants with ARIs who had been enrolled in a prospective, community‐based, surveillance cohort from Northern Manhattan, New York City.^8^ The current study assessed the clinical and socioeconomic burden associated with RV/EV among participants. Briefly, following written informed consent, participants underwent surveillance for ARI symptoms from December 2012 to September 2017. Eligible households had three or more members with at least one member under 18 years of age, were Spanish‐ or English‐speaking, and had a household reporter with a cellular telephone with text messaging capacity who reported ARIs in household members to the study team, as previously described.^8^ The Columbia University Irving Medical Center (CUIMC) institutional review board approved the current retrospective study with a waiver of documentation of informed consent.

### 2.2 Identifying ARIs and assessing ARI burden

To identify potential ARIs, the study team sent text messages twice weekly to the household reporter inquiring about ARIs among their household members.^8^ The study team collected nasal swabs from ill participants in their homes, generally within 2 days of reported symptoms, if at least two of the following ARI symptoms were reported: fever (defined as ≥37.8°C)/feverishness, runny nose/ congestion, sore throat, cough, chills, headache, wheezing, and/or myalgia. In addition, nasal swabs were collected from infants less than 1 year of age if they only had runny nose/congestion.

To assess the clinical and socioeconomic burden associated with detection of RV/EV, the study team called the household reporter starting 10 days after the onset of ARI symptoms. Data collected during these calls included: duration of ARI symptoms; days of missed school or work for ill participants or their caregivers; seeking medical care for the ARI in primary care clinics, urgent care, or emergency departments (ED); reported diagnosis such as pharyngitis, otitis, pneumonia, sinusitis; hospitalizations; and use of antibiotics.

### 2.3 Diagnostic testing for respiratory pathogens

Nasal swab samples were tested for respiratory pathogens in a research laboratory at CUIMC using multiplex RT‐polymerase chain reaction (PCR) (FilmArray Panel, BioFire Diagnostics, Inc. Salt Lake City, Utah). This assay identified the following respiratory pathogens: adenovirus, human coronaviruses (types HKU1, NL63, 229E, OC43), human metapneumovirus, RV/EV, influenza (types A, A/H1, A/H3, A/H1–2009, B), parainfluenza (types 1, 2, 3, 4), RSV, *Bordetella pertussis*, *Chlamydophila pneumoniae*, and *Mycoplasma pneumoniae*.^11^ The assay has a reported 95.7% sensitivity and 94.6% specificity for RV/EV.^12^

### 2.4 Analysis

We assessed detection of RV/EV among participants in three age groups: <5, 5–17, and ≥18 years of age. To describe seasonal trends, epidemiological curves of ARIs with RV/EV detected were created for children ≤17 years versus adults ≥18 years of age. Demographic, clinical (selected comorbidities), and socioeconomic characteristics (e.g., education, employment status, and insurance type) of participants with RV/EV who did and did not seek medical care were compared using simple logistic regression. The clinical and socioeconomic burden among those who did and did not seek medical care and among those with RV/EV alone vs. RV/EV co‐detected with another respiratory pathogen were compared by Student t‐test, chi‐square, and Wilcoxon‐rank test, as appropriate. We used multiple logistic regression to investigate characteristics of those who did and did not seek medical care and the burden among those with and without co‐detection while controlling for selected characteristics found to be significantly associated (*p* < 0.05) with seeking medical care in the bivariate analysis. Analyses were conducted using STATA version 14 (STATACorp. College Station, TX).

## 3 RESULTS

**FIGURE 1:**
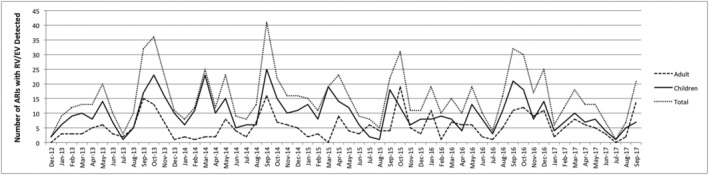
Seasonal epidemiology of rhinoviruses/enteroviruses (RV/EV) detection associated with ARIs. The number of acute respiratory infections (ARIs) with RV/EV detected monthly in adults ≥18 years old, children ≤17 years old, and all participants are shown from December 2012 to September 2017 with peaks in early spring and early fall

During the study period, 405 primarily immigrant and Latino households consisting of 1915 household members were recruited to the prospective surveillance study. Participants experienced 3016 ARIs during the study period that met the ARI case definition and 2756 (91.4%) had a swab obtained. Among the 1617 (58.7%) samples with a respiratory pathogen detected, RV/EV were detected in 885 (54.7%) participants. RV/EV were detected throughout the year with annual peaks in early Spring and early Fall (Figure 1).

### 3.1 Burden of illness associated with RV/EV

Selected characteristics of the 885 participants with RV/EV detected are shown in Table 1. Most participants with RV/EV detected were female (59.1%), ≤17 years old (64.2%), and Hispanic (99.7%). The median age of adults was 41.6 years (IQR 29.5–50.8 years) and 2.1% were 65 years of age and older. Among adults, 85.2% (270/317) were female and among those ≤17 years old, 44.5% (253/568) were female.

**TABLE 1:** Characteristics of participants with rhinovirus/enterovirus detected who did and did not seek medical care, bivariate analysis

Participant characteristics, *n* (%)^a^	All *N* = 885	Did seek medical care *N* = 199	Did not seek medical care *N* = 686	Odds ratio (95% confidence interval)	*P* value
Sex					
Female	523 (59.1%)	99 (49.7%)	424 (61.8%)		
Male	362 (40.9%)	100 (50.3%)	262 (38.2%)	1.63 (1.19–2.24)	0.002
Age (in years)					
≥18	317 (35.8%)	35 (17.6%)	282 (41.1%)	Reference	
5–17	319 (36.0%)	80 (40.2%)	239 (34.8%)	4.10 (2.64–6.36)	<0.001
<5	249 (28.1%)	84 (42.2%)	165 (24.0%)	2.69 (1.75–4.16)	<0.001
Chronic respiratory conditions	178 (20.1%)	49 (24.6%)	129 (18.8%)	1.41 (0.97–2.05)	0.074
Chronic non‐respiratory conditions	210 (23.7%)	33 (16.6%)	177 (25.8%)	0.57 (0.38–0.86)	0.008
Insurance					
Private	123 (13.9%)	25 (12.6%)	98 (14.4%)	Reference	
Medicaid/public	703 (79.9%)	169 (84.9%)	534 (78.5%)	1.24 (0.77–1.99)	0.371
Uninsured	53 (6.0%)	5 (2.5%)	48 (7.1%)	0.41 (0.15–1.13)	0.085

^a^ Column percentages are shown.

Medical care was sought by 22.5% (199/885) of participants, including 33.7% (84/249) of children <5 years old, 25.1% (80/319) of children 5–17 years old, and 11.0% (35/317) of adults. Overall, participants who sought medical care were more likely to be male and ≤17 years of age and less likely to have a chronic non‐respiratory condition (Table 1). Among adults, neither employment status (OR: 1.25 [95% CI: 0.59–2.66], *p* = 0.553) nor level of education (OR: 0.84 [95% CI: 0.41–1.73], *p* = 0.634) was associated with seeking medical care. Among children, daycare/school attendance vs. home care was not associated with seeking medical care (OR: 0.91 [95% CI: 0.60–2.38], *p* = 0.667). In multiple logistic regression, only age ≤17 years remained associated with seeking medical care (aOR: 2.96 [95% CI: 1.88–4.66], *p* < 0.001).

Overall, 23.5% (208/885) of participants with ARIs associated with RV/EV missed at least 1 day of work or school and 10.5% (93/885) used antibiotics (Table 2) including 8.2% of adults and 11.8% of children (*p* = 0.09). Participants who sought medical care had significantly more days of illness (7 vs. 5 days) and participants/their caretakers were more likely to miss school and/or work (47.7% vs. 16.4%). Of the 199 participants who sought medical care, 68 (34.2%) were prescribed antibiotics including 36.9%, 33.8%, and 28.6% of those <5, 5–17, and ≥18 years of age who sought care, respectively; participants <5 years of age were not more likely to be prescribed antibiotics than those ≥18 years of age (*p* = 0.44). Fifty‐five reported receiving antibiotics for the following diagnoses: pharyngitis, otitis media, bronchitis, sinusitis, pneumonia, and/or an asthma exacerbation. Among the 686 participants who did not seek medical care, 25 (3.6%) used antibiotics, most of whom were adults (16/25, 64.0%).

**TABLE 2:** Clinical and socioeconomic burden associated with rhinovirus/enterovirus detected in participants who did and did not seek medical care

Burden of illness	All *N* = 885	Did seek medical care *N* = 199	Did not seek medical care *N* = 686	Odds ratio (95% confidence interval)	*P* value
Days of illness, Median (IQR)	5 (2,10)	7 (3,11)	5 (2,9)	NA	0.005^a^
Missed ≥1 day work/school, *n* (%)^b^	208 (23.5%)	95 (47.7%)	113 (16.4%)	4.6 (3.22–6.38)	<0.001
Antibiotic use, *n* (%)	93 (10.2%)	68 (34.2%)	25 (3.6%)	13.72 (8.36–22.52)	<0.001

*Note*: Abbreviations used in the table: IQR—interquartile range.

^a^ Wilcoxon signed‐rank test.

^b^ Five participants had missing data for missing work/school.

### 3.2 Burden of illness associated with co‐detection of another respiratory pathogen

Co‐detection of another respiratory pathogen occurred in 8.0% (*n* = 70) of ARIs with RV/EV detected. The following were co‐detected: coronaviruses (*n* = 22), parainfluenza (*n* = 12), human metapneumovirus (*n* = 9), RSV (*n* = 9), adenovirus (*n* = 7), *M. pneumoniae* (*n* = 5), influenza (*n* = 4), and *C. pneumoniae* (*n* = 2). Most co‐detections (81.4%) occurred in children. The proportion of participants who reported symptoms of runny nose/congestion, cough, sore throat, and muscle/body aches was similar in those with and without co‐detection (data not shown). However, fever/feverishness was reported more frequently in those with co‐detection than without co‐detection (44.3% vs. 31.6%, respectively, *p* = 0.031). Those with and without co‐detection had a similar duration of illness and similar rates of missing work/school or antibiotics use (Table 3). While bivariate analysis found that participants with co‐detection were more likely to seek medical care, in multiple regression analysis, co‐detection was not significantly associated with seeking medical care (aOR: 1.52 [95% CI: 0.88–2.68], *p* = 0.126).

**TABLE 3:** Clinical and socioeconomic burden associated with rhinovirus/enterovirus (RV/EV) detected versus RV/EV co‐detected with another respiratory pathogens

Burden of illness	RV/EV only *N* = 815	RV/EV co‐detected with another pathogen *N* = 70	Odds ratio (95% confidence interval)	P value
Days of illness Median (IQR)	5 (2, 10)	6.5 (3, 10)	NA	0.179^a^
Missed ≥1 day work/school, *n* (%)^b^	190 (23.4%)	18 (26.1%)	1.15 (0.66–2.02)	0.618
Sought medical care, *n* (%)	176 (21.6%)	23 (32.9%)	1.77 (1.05–3.01)	0.030
Antibiotic use, *n* (%)	83 (10.2%)	10 (14.3%)	1.47 (0.72–2.98)	0.286

*Note*: Abbreviations used in the table: IQR—interquartile range.

^a^ Wilcoxon signed‐rank test.

^b^ Five participants had missing data for missing work/school.

## 4 DISCUSSION

This community‐based surveillance study provides insights into the burden of ARIs associated with RV/EV for both medically‐attended and non‐medically‐attended events as most (77.5%) participants did not seek medical care. While the clinical and socioeconomic burden of ARIs associated with RV/EV detection was higher among those who sought care, participants who did not seek care had a median of 5 days of illness and 16% missed at least 1 day of work and/or school. While direct healthcare costs associated with RV/EV have not been measured, both direct healthcare and indirect costs associated with non‐influenza viral infections are estimated to be $40 billion per year in the United States; this is greater than the costs associated with chronic diseases including hypertension, asthma, or chronic obstructive pulmonary disease.^5^ We observed another consequence of RV/EV; most individuals with symptomatic ARIs continued to attend school or work and thus were potentially infecting others.

Seeking medical care was associated with antibiotic use in both adults and children. While some participants received antibiotics from their providers to treat other diagnosis, for example, otitis media and pneumonia, antibiotic use for symptoms consistent with viral illness highlights the need to improve antimicrobial stewardship in ambulatory settings. Globally, 85%–95% of all antibiotics are prescribed for outpatients including those cared for in urgent care facilities, clinics, and EDs.^13^ In 2017, approximately 249 million oral antibiotic prescriptions, equivalent to 763 prescriptions per 1000 persons, were dispensed from community pharmacies in the United States and those 20 years of age and older had highest prescription rates than younger individuals.^14^ It has been estimated that at least 30% of the prescribed antibiotics in the outpatient settings are unnecessary and prescriptions for ARIs are the most reason for prescriptions.^15^ Furthermore, in the current study, 3.6% of participants, more often adults, who did not seek medical care, used antibiotics. As other studies have shown, adults are more likely to self‐prescribe or obtain antibiotics from non‐traditional settings such as bodegas, which are common in the study community.^16^

Several of our findings corroborated those described previously. More than half of the ARIs with RV/EV detected occurred in children, consistent with previous observations that RV/EV are the most common causes of ARI in children.^17^ More than half of the ARIs associated with RV/EV occurred in females, but the majority of participants were female and likely had more contact with children; additionally, 25% of females worked within the home. As previously described, RV/EV occurred year‐round with seasonality peaks in early spring and early fall.^1^, ^3^, ^18^

Co‐detection of another respiratory pathogen with RV/EV occurred in only 8% of ARIs. While those with co‐detection were more likely to report fever/feeling feverish, the clinical symptoms, days of illness, and socioeconomic burden were comparable in those with RV/EV alone versus RV/EV co‐detected with another pathogen, further confirming the substantial burden and clinical impact of RV/EV. As previously described, we also demonstrated that co‐detection was more common in children.^19^, ^20^ This could be due to increased exposure, age‐related susceptibility, less effective hygienic practices, and/or prolonged RV/EV shedding in children. The clinical significance of co‐detection/co‐infection is uncertain. Some studies have found that viral co‐detection is associated with worse clinical outcomes^21^; others have found less severe disease,^22^ while others have found no impact of co‐detection.^20^, ^23^, ^24^ Outcomes appear to vary by specific patterns of co‐detection.^20^ Some studies have demonstrated that RV renders the host less likely to be co‐infected.^25^, ^26^ For example, the relationship between RSV and RV may be antagonistic as infection by one temporarily reduces acquisition of the other virus.^26^, ^27^ Furthermore, co‐detections may represent “false positive” results as RT‐PCR assays may be detecting non‐viable viruses.^9^, ^10^

This study has limitations. It may lack generalizability as it was performed in New York City with predominantly Hispanic/Latino participants. The burden of illness was reported by a household reporter; ill participants did not provide additional verification. The proportion of participants who missed work/school and symptom duration may be underestimated due to recall bias. Burden may also be underestimated as some adults did not work outside the home as they were unemployed or worked as homemakers. The use of RT‐PCR may overestimate co‐detection due to prolonged viral shedding and/or detection of non‐viable virus. Finally, the RT‐PCR assay used in this study does not distinguish RV from EV so differences associated with different RV/EV types could not be assessed.

## 5 CONCLUSIONS

This study provides further evidence of the clinical and socioeconomic burden associated with RV/EV in both adults and children. We noted that most participants with RV/EV did not seek medical care. Most did not miss work or school and thus, likely infected others outside the home. We found inappropriate antibiotic use among participants, which supports strengthening antimicrobial stewardship efforts in ambulatory settings. Further research could help assess the socioeconomic burden of RV/EV in other patient populations and prevention strategies for RV/EV should be developed.

## AUTHOR CONTRIBUTIONS

**Katia Halabi:** Data curation; formal analysis; investigation; methodology; project administration. **Melissa Stockwell:** Conceptualization; formal analysis; funding acquisition; investigation; methodology; validation. **Luis Alba:** Data curation; formal analysis; methodology. **Celibell Vargas:** Data curation; formal analysis; investigation; methodology. **Carrie Reed:** Conceptualization; methodology. **Lisa Saiman:** Conceptualization; formal analysis; funding acquisition; investigation; methodology; project administration; supervision; validation.

### PEER REVIEW

The peer review history for this article is available at https://publons.com/publon/10.1111/irv.12989.

## Data Availability

The data that support the findings of this study are available from the corresponding author upon reasonable request.

## References

[1] Royston L , Tapparel C . Rhinoviruses and respiratory enteroviruses: not as simple as ABC. Viruses. 2016;8(1):16. doi:10.3390/v8010016 PMC4728576 26761027

[2] To KKW , Yip CCY , Yuen KY . Rhinovirus ‐ from bench to bedside. J Formos Med Assoc. 2017;116(7):496‐504. doi:10.1016/j.jfma.2017.04.009 28495415

[3] Asner SA , Petrich A , Hamid JS , Mertz D , Richardson SE , Smieja M . Clinical severity of rhinovirus/enterovirus compared to other respiratory viruses in children. Influenza Other Respi Viruses. 2014;8(4):436‐442. doi:10.1111/irv.12255 PMC4181803 24801963

[4] Spaeder MC , Custer JW , Miles AH , et al. A multicenter outcomes analysis of children with severe rhino/enteroviral respiratory infection. Pediatr Crit Care Med. 2015;16(2):119‐123. doi:10.1097/PCC.0000000000000308 25647121

[5] Fendrick AM , Monto AS , Nightengale B , Sarnes M . The economic burden of non‐influenza‐related viral respiratory tract infection in the United States. Arch Intern Med. 2003;163(4):487‐494. doi:10.1001/archinte.163.4.487 12588210

[6] Miller EK , Linder J , Kraft D , et al. Hospitalizations and outpatient visits for rhinovirus‐associated acute respiratory illness in adults. J Allergy Clin Immunol. 2016;137(3):734‐743. doi:10.1016/j.jaci.2015.06.017 26255695 PMC4744574

[7] Fine J , Bray‐Aschenbrenner A , Williams H , Buchanan P , Werner J . The resource burden of infections with rhinovirus/enterovirus, influenza, and respiratory syncytial virus in children. Clin Pediatr (Phila). 2019;58(2):177‐184. doi:10.1177/0009922818809483 30387696

[8] Stockwell MS , Reed C , Vargas CY , et al. Five‐year community surveillance study for acute respiratory infections using text messaging: findings from the MoSAIC study. Clin Infect Dis. 2022;Jan 17. Online ahead of print. doi:10.1093/cid/ciac027 PMC9383201 35037056

[9] Zlateva KT , de Vries JJ , Coenjaerts FE , et al. Prolonged shedding of rhinovirus and re‐infection in adults with respiratory tract illness. Eur Respir J. 2014;44:169‐177. doi:10.1183/09031936.00172113, 1 24876172

[10] Jartti T , Lehtinen P , Vuorinen T , Koskenvuo M , Ruuskanen O . Persistence of rhinovirus and enterovirus RNA after acute respiratory illness in children. J Med Virol. 2004;72(4):695‐699. doi:10.1002/jmv.20027 14981776

[11] Biofire FilmArray System Documents . ‐Biofire Filmarray Respiratory Panel instruction booklet. https://www.biofiredx.com/support/documents/-toggle-id-3. Accessed June 14th, 2021.

[12] Biomerieux‐Diagnostics . https://www.biomerieux-diagnostics.com/filmarrayr-respiratory-panel. Accessed August 7, 2020.

[13] King LM , Fleming‐Dutra KE , Hicks LA . Advances in optimizing the prescription of antibiotics in outpatient settings. BMJ. 2018;363:k3047. doi:10.1136/bmj.k3047 30420401 PMC6511972

[14] Centers for Disease Control and Prevention . Outpatient antibiotic prescriptions—United States, 2017. https://www.cdc.gov/antibiotic‐use/community/programs‐measurement/state‐local‐activities/outpatient‐antibiotic‐prescriptions‐US‐2017.html. Accessed July 16, 2020.

[15] Fleming‐Dutra KE , Hersh AL , Shapiro DJ , et al. Prevalence of inappropriate antibiotic prescriptions among us ambulatory care visits, 2010‐2011. Jama. 2016;315(17):1864‐1873. doi:10.1001/jama.2016.4151 27139059

[16] Larson El DJ , Garcia M , Smolowitz J Factors which influence Latino community members to self‐prescribe antibiotics. Nurs Res. 2006;55:94‐102. doi:10.1097/00006199-200603000-00004, 2 16601621

[17] Kieninger E , Fuchs O , Latzin P , Frey U , Regamey N . Rhinovirus infections in infancy and early childhood. Eur Respir J. 2013;41(2):443‐452. doi:10.1183/09031936.00203511 22743674

[18] Winther B , Hayden FG , Hendley JO . Picornavirus infections in children diagnosed by RT‐PCR during longitudinal surveillance with weekly sampling: association with symptomatic illness and effect of season. J Med Virol. 2006;78(5):644‐650. doi:10.1002/jmv.20588 16555289

[19] Peng D , Zhao D , Liu J , et al. Multipathogen infections in hospitalized children with acute respiratory infections. Virol J. 2009;6(1):155. doi:10.1186/1743-422X-6-155 19788746 PMC2762469

[20] Zimmerman RK , Rinaldo CR , Nowalk MP , et al. Viral infections in outpatients with medically attended acute respiratory illness during the 2012‐2013 influenza season. BMC Infect Dis. 2015;15(1):87. doi:10.1186/s12879-015-0806-2 25887948 PMC4344779

[21] Cilla G , Oñate E , Perez‐Yarza EG , Montes M , Vicente D , Perez‐Trallero E . Viruses in community‐acquired pneumonia in children aged less than 3 years old: high rate of viral coinfection. J Med Virol. 2008;80(10):1843‐1849. doi:10.1002/jmv.21271 18712820 PMC7166914

[22] Martin ET , Kuypers J , Wald A , Englund JA . Multiple versus single virus respiratory infections: viral load and clinical disease severity in hospitalized children. Influenza Other Respi Viruses. 2012;6(1):71‐77. doi:10.1111/j.1750-2659.2011.00265.x PMC3175338 21668660

[23] Asner SA , Rose W , Petrich A , Richardson S , Tran DJ . Is virus coinfection a predictor of severity in children with viral respiratory infections? Clin Microbiol Infect. 2015;21(3):264, e1–e6. doi:10.1016/j.cmi.2014.08.024 25596778 PMC7128494

[24] Cebey‐Lopez M , Herberg J , Pardo‐Seco J , et al. Does viral co‐infection influence the severity of acute respiratory infection in children? PLoS One. 2016;11(4):e0152481. doi:10.1371/journal.pone.0152481 27096199 PMC4838299

[25] Debiaggi M , Canducci F , Ceresola ER , Clementi M . The role of infections and coinfections with newly identified and emerging respiratory viruses in children. Virol J. 2012;9(1):247. doi:10.1186/1743-422X-9-247 23102237 PMC3573994

[26] Greer RM , McErlean P , Arden KE , et al. Do rhinoviruses reduce the probability of viral co‐detection during acute respiratory tract infections? J Clin Virol. 2009;45(1):10‐15. doi:10.1016/j.jcv.2009.03.008 19376742 PMC7185458

[27] Karppinen S , Toivonen L , Schuez‐Havupalo L , Waris M , Peltola V . Interference between respiratory syncytial virus and rhinovirus in respiratory tract infections in children. Clin Microbiol Infect. 2016;22:208, e1–e208, e6. doi:10.1016/j.cmi.2015.10.002, 2 26482269

